# The Combined Application of Impinger System and Permeation Tube for the Generation of Volatile Organic Compound Standard Gas Mixtures at Varying Diluent Flow Rates

**DOI:** 10.3390/s120810964

**Published:** 2012-08-08

**Authors:** Ki-Hyun Kim, Janice Susaya, Jinwoo Cho, David Parker

**Affiliations:** 1 Department of Environment & Energy, Sejong University, Seoul 143-747, Korea; E-Mails: jsusaya@gmail.com (J.S.); jinwoocho@sejong.ac.kr (J.C.); 2 College of Agriculture, Science and Engineering, West Texas A&M University, Canyon, TX 79016, USA; E-Mail: dparker@mail.wtamu.edu

**Keywords:** impinge, permeation, benzene, toluene, ethylbenzene, xylene

## Abstract

Commercial standard gas generators are often complex and expensive devices. The objective of this research was to assess the performance of a simplified glass impinger system for standard gas generation from a permeation tube (PT) device. The performance of the impinger standard gas generation system was assessed for four aromatic VOCs (benzene, toluene, ethylbenzene, and *m*-xylene; BTEX) at varying flow rates (FR) of 50 to 800 mL·min^−1^. Because actual permeation rate (APR) values deviated from those computed by the manufacturer's formula (MPR), new empirical relationships were developed to derive the predicted PR (PPR) of the target components. Experimental results corrected by such a formula indicate that the compatibility between the APR and MPR generally increased with low FR, while the reproducibility was generally reduced with decreasing flow rate. Although compatibility between different PRs is at a relatively small and narrow FR range, the use of correction formula is recommendable for the accurate use of PT.

## 1. Introduction

The Blacksmith Institute World's Worst Polluted Places report designated indoor air pollution and urban air quality as two of the World's worst pollution problems in 2006 and 2007, respectively [1]. It is reported that 2.4 million people die each year from causes directly attributable to air pollution, while 1.5 million of those are due to indoor air pollution [2]. To control pollutant emissions from various sources, regulatory guidelines are now in effect in many developed as well as developing countries. As a result, demand is on the rise for accurate assessment of pollutant concentrations.

An impinger system has been a common choice for sorptive sampling, especially trapping of gaseous pollutants in a liquid sorbent media [3,4]. The use of an impinger system was also extended to various applications, e.g., estimation of emission rates of various odorants from sediment samples collected from a polluted lake environment [5].

It is acknowledged that the use of permeation tube (PT) device is precise and repeatable enough to generate National Institute of Standards and Technology (NIST)-traceable calibration gas standards, over long-term periods, with concentrations ranging from part per trillion (ppt) to high part per million (ppm) [6]. The calibrant chemical permeates through the polymeric walls of the tube for a constant rate at a given temperature. According to the manufacturer's guide, one can generate standard gas for calibration for each target compound by controlling the temperature and the matrix gas flowing through a PT device. Hence, PT is commonly used for standard gas generation with the aid of a reliable gas generator equipped with temperature controlling system (e.g., dynacalibrator).

In our recent studies, we explored the performance of commercially available dynacalibrator/PT devices in relation to changing flow rate of diluent [7] and across varying temperature [8]. The results of our recent studies were helpful in defining the suitability of the PT method with these precise devices designed specifically for such application. For the purpose of our study, we selected four aromatic volatile compounds (VOC) which include benzene (B), toluene (T), ethylbenzene (E), and *m*-xylene (X). As the analytical method of these compounds is well defined, we selected them as the main target components of our study. The objective of this research was to assess the performance of a simplified standard gas generation system constructed from a glass impinger and permeation tube (PT) device.

## 2. Methods

### 2.1. Configuration of the PT-Impinger System for Standard Gas Generation

**Figure 1.**
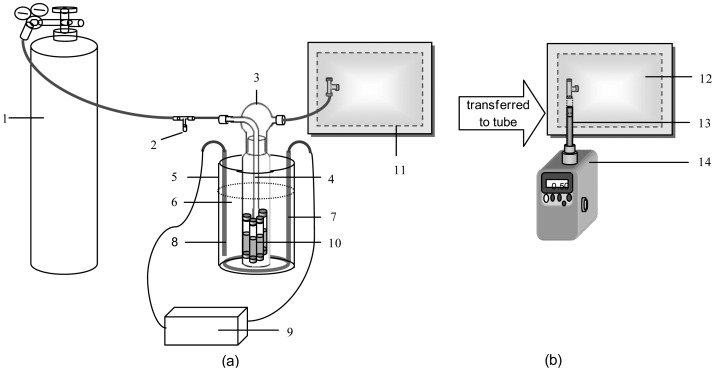
Illustration of (**a**) the sampling device for generating gaseous BTEX standards using an impinger system and (**b**) the secondary sampling system for transferring BTEX samples from the Tedlar bag into the sorbent tube for GC-FID determination. Number labels: (1) Pure N_2_ tank; (2) N_2_ flow regulator; (3) Impinger bottle and cap with inlet (left side) and outlet (right side) ports (750 mL); (4) Glass tubing with a bubbler tip to evenly distribute the diluent gas; (5) Aluminum container; (6) Water heated to 25 °C; (7) Heater; (8) Temperature sensor; (9) Temperature regulator; (10) Permeation tube device for BTEX; (11) Empty 10 L Tedlar bag; (12) Tedlar bag containing BTEX; (13) Sorbent tube; and (14) Sibata vacuum pump.

Dynacal permeation tube devices for four target VOCs [benzene (B), toluene (T), ethylbenzene (E), and m-xylene (X))] were purchased from Valco Instruments Co. Inc. (VICI Metronics Inc, Poulsbo, WA, USA). The manufacturer-published permeation rate (PR) values at each reference temperature were 16,000 (at 70 °C), 18,849 (at 80 °C), 21,774 (at 100 °C), and 21,714 ng·min^−1^ (at 100 °C), respectively. These same PT devices were used for our recent study for standard gas generation using a more complicated commercial dynacalibrator system [7,8]. The basic information of target compounds (e.g., structural formula, density, molecular weight, and CAS no.) and PT device (e.g., sales order no., part no., type, and PR at a certain temperature) is presented in Table 1. For this experiment, gaseous BTEX was generated at constant temperature by placing PT devices in an impinger system (750 mL volume; 6.5 cm diameter; and 24.5 cm height up to glass rim, Figure 1).

The PT devices were wiped clean and placed inside the impinger bottle using forceps. Glass impingers used in this study were thoroughly washed and rinsed with distilled water and kept away from possible sources of contamination (e.g., dust) prior to use. In the absence of carrier flow, the impinger chamber and surrounding compartments can be contaminated with target components; hence we ensured the carrier flow was maintained continuously [9]. The impinger bottle was immersed in a water bath, and kept at 25 °C using a digital temperature controller Figure 1(a). The system became stabilized at the selected temperature after approximately one hour [10,11].

### 2.2. Sampling and Calibration of PT-Generated Standard Gas

The impinger system was flushed with an inert carrier gas (ultrapure N_2_) at a steady flow. As supplied at the inlet of the impinger system, N_2_ gas sweeps the target gas/vapor from the impinger glass (Figure 1(a)). A maximum flow rate for this experiment was set at 800 mL·min^−1^ by considering the back-pressure that builds up in the impinger bottle to balance between several parameters (e.g., impinger volume capacity, tubing size, *etc.*). The vertical tubular column (with a bubbler at the bottom tip of the impinger caps) served to supply N_2_ gas evenly inside the impinger.

Gaseous standards were generated at six different FR (50 to 800 mL·min^−1^). An initial short flushing time was given prior to each run to saturate the impinger system. The standard gas streams exiting the impinger system thru its outlet port were collected in the 10 L Tedlar bags. The Tedlar bags were flushed several times with ultra-pure N_2_ prior to sample collection and were subjected to bag blank analysis to assure they were clean. Upon collection of the samples, Tedlar bags were kept at room temperature inside black bags until analysis (within 24 h). Once BTEX samples were collected in Tedlar bags, the second phase of the experiment was carried out by transferring the air samples into the sorbent tube at a flow rate of 0.2 L·min^−1^ using a portable vacuum pump (Sibata MP Σ300, Soka-City, Saitama, Japan). Sorbent tubes were loaded into the Ultra-TD autosampler (Model: Unity, Markes International, Llantrisant, UK) to induce thermal desorption for the GC-FID analysis (Varian 450-GC, Walnut Creek, CA, USA). The instrumental settings for this tube-based analysis are provided in Table 2. The stainless steel sorbent tubes were packed with 300 mg of carbopack-X sorbent material. All sorbent tubes were pre-conditioned before use. As samples taken at low FR had significantly high concentrations, e.g., actual measured concentration (AC) of 398 ng·mL^−1^ for benzene at FR of 50 mL·min^−1^, the tube-based analysis of samples taken at this flow rate was made after one-step dilution (e.g., 2-fold reduction). This extra step was necessary to adjust a target analyte concentration to fit into the common detection range of the instrumental setups for this study.

### 2.3. Cylinder Gas Standard Calibration for BTEX as a Main Reference

As a means to determine the actual concentration of PT-generated standard gas, an ancillary cross calibration was conducted by using a certified standard gas purchased separately in cylinder tanks (Rigas, Dae Jeon, Korea). The mixture of 6 ppm standard was prepared by injecting 300 mL of 20 ppm BTEX (using a gas-tight syringe) into a 1 L Tedlar bag partially filled with 700 mL of ultra-pure N_2_. This bag was then kept inside another black plastic bag and left to stabilize for 10 to 20 mins. These samples were then analyzed quickly to avoid sorptive loss due to extended storage [12]. The cylinder-based standards were also analyzed by following the above-described procedures for PT-generated samples Figure 1(b). For these gaseous standards, experimental conditions for calibration analysis are listed in Table 3. Hence, the actual concentrations of PT-generated gases can be estimated against certified standard gas in cylinder. Method detection limits (MDL) for each compound were determined prior to the main experiments. The detection limits for B, T, X, and E were estimated as 1.2, 2.7, 0.8, and 0.30 ng in absolute mass term, respectively. If 1 L of sample is loaded onto a sorbent tube, those detection limit values equate to 0.36, 0.72, 0.22, and 0.10 ppb, respectively (note that the slope of ethylbenzene was estimated based on carbon and slope ratio of other compounds due to the unavailability of its gaseous primary standard in this study). Linearity of the system was excellent within the selected calibration range with R^2^ values greater than 0.99 for all compounds. The precision, if expressed in terms of relative standard error (RSE) values, was excellent at <1% for all compounds.

### 2.4. Adjustment of PR between Varying Temperatures and the Resulting Concentrations of PT-Generated Standard

Knowing that the PR values of each target compound can be different from the manufacturer's recommendation and the actual measurements, we defined the three PR terms as follows: (1) manufacturer's PR (MPR), (2) actual PR (APR) based on the GC-based quantification of target compounds, and (3) predicted PR (PPR) by building empirical equation from the APR values. Derivation of each PR term can be obtained as follows:

According to the manufacturer's guide, the MPR values are generally defined for a given temperature (Table 1). For our experimental conditions (T = 25 °C), the MPR values can be approximated against reference temperature (T_o_) by the equation [13]:

(1) $$\log\;\text{P}=\text{log}\;{\text{P}}_{\text{o}}+0.034\;(\text{T}-{\text{T}}_{\text{o}})$$

where:

P_o_ = PR at a reference temperature (T_o_)
P = PR at adjusted temperature (T)

In this study, experimental temperature was set at 25 °C to slightly exceed room temperature. This condition is thus expected to be comparable to the lower bound temperature limit (30 °C) for PT calibration commonly recommended by the manufacturer [14]. Investigation of the actual effect of changing temperature on PR values for the simplified system was carried out [8]. According to equation 1, the new PR values adjusted for our experimental conditions were 472 (B), 254 (T), 61.4 (E), and 61.2 ng·min^−1^ (X) at 25 °C (Table 1). The expected concentrations of PT-generated gases were computed using the manufacturer-published equation (MC) with new PR values under given FR:

(2) MC=(PR×K)/F

where:

MC = manufacturer-given concentration of gas standard in ppm
PR = Permeation rate (ng·min^−1^)
K = molar constant (RT/molecular weight of gas)
F = diluent flow rate (mL·min^−1^)

For comparison, experimentally determined concentrations of BTEX from PT-generated samples were defined as the actual concentrations (AC) and used to derive the corresponding APR values. For the derivation of APR, the total amount of each target compound contained in each 10-L Tedlar bag was first estimated from the experimentally determined concentration. Then, this estimated amount (mass in ng) was divided by the total duration of sampling (min) to derive APR in ng·min^−1^. Finally, equations derived from AC-FR plots were used to derive predicted concentrations (PC) and the corresponding PPR of each compound at specific FR.

## 3. Results and Discussion

### 3.1. Actual Concentration (AC) of PT-Generated Gases

**Figure 2.**
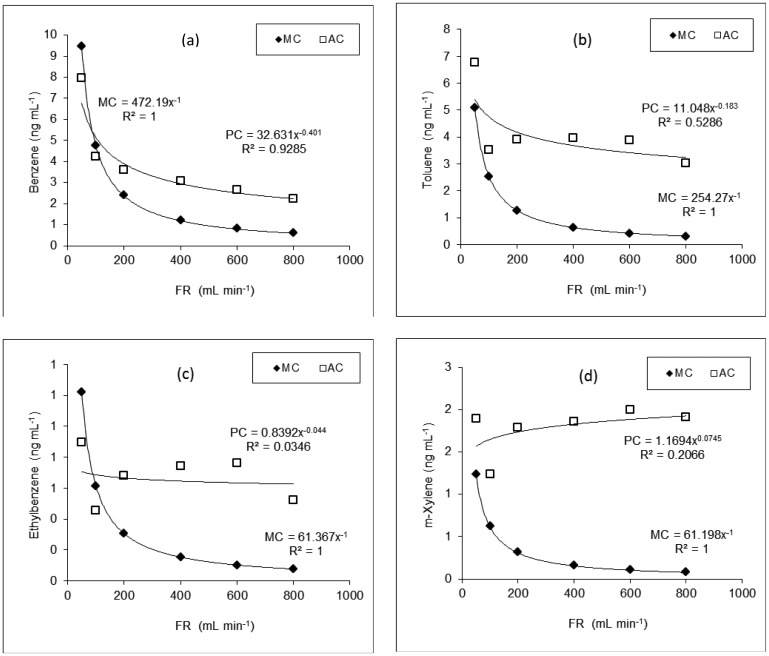
Comparison of BTEX data (ng·mL^−1^) between the AC and MC as a function of diluent flow rate (mL·min^−1^). Labels: MC = manufacturer given concentration; AC = actual measured concentration; and PC = predicted concentration using our developed equations.

As described above, we already defined three different types of concentration terms such as MC, AC, and PC. Because of differences in concentration data derived between different terms, their values are presented to allow comparison with each other (Table 4). Plots of AC values for each compound against FR exhibited curvilinear patterns (Figure 2). A strong inverse relationship between AC and diluent FR was observed for benzene (R^2^ = 0.9285). However, the relationships for T, E, and X were not as strong, with R^2^ values of 0.5286, 0.0346, and 0.2066, respectively. These measured concentration (AC) values of BTEX were compared with those calculated using equations of the manufacturer (MC) at each specific flow rate at 25 °C. In case of B and E, AC values were comparable with MC at lower FR (e.g., <100 mL·min^−1^) but deviated considerably with increasing FR (Figure 2). However, the pattern of deviation between MC and AC changed greatly from each other.

The reproducibility of our PT-impinger combination for the gas standard generation was assessed from triplicate analyses of AC in terms of relative standard error (RSE (%), Table 4). Comparison of RSE data for all compounds generally indicates that reproducibility generally increases with decreasing flow rates, although there is a systematic exception at 600 mL·min^−1^. It is interesting to find the poorest RSE values with the lowest FR range (50 mL·min^−1^, Table 4).

### 3.2. Comparison between Different PR Values as a Function of Flow Rate

**Figure 3.**
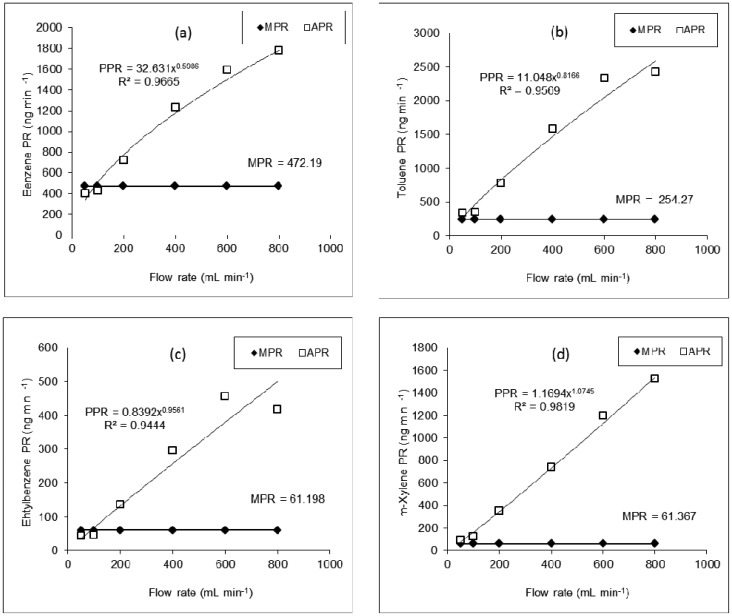
Dynamic relationship between the measured permeation rate (PR: ng·min^−1^) and flow rates (FR: mL·min^−1^) of dilution gas by using impinger system. Labels: MPR = manufacturer given PR; APR = actual measured PR; and PPR = predicted PR using our developed equations.

Based on the manufacturer's given equation, the PR value for a given compound should remain constant at varying diluent FR at any given temperature (Table 4). Hence, to assess the effect of flow rate change on the PR values of BTEX, APR values derived from the corresponding AC data sets were plotted against FR (Figure 3).

According to our evaluation of APR-FR plots for all compounds, these parameters tend to maintain a curvilinear trend in which APR values increase with increasing flow rates. It shows that the compatibility between APR and MPR tends to decrease systematically with increasing FR. The breadth of difference between the measured (APR) and the given permeation rates (MPR), if expressed in terms of percent differences, is systematic and becomes fairly large at the far extreme FR conditions (Figure 3). The maximum compatibility between APR and MPR is thus found at either 50 or 100 mL·min^−1^ (Table 4). It is thus unrealistic to use uniform PR values as defined by the manufacturer [13] across all FR values. Therefore, it may be reasonable to conclude that the PR values of a specific compound should be determined in a tight relationship with FR at a given temperature. Hence, our efforts were directed towards the establishment of an alternative equation to better predict the PR of PT devices.

### 3.3. Establishment of Equations for the Prediction of PR

By plotting actual permeation rate (APR) values as a function of FR (Figure 3), we derived the best fit regression equation models to yield the predicted permeation rate (PPR) at any FR (and a given temperature). Several regression models (*i.e.*, logarithmic, exponential, polynomial, power, *etc.*) were tested by using some statistical software packages and the graphing features (e.g., Microsoft Excel). By employing the coefficient of determination (R^2^) as the key selection criteria, the power regression model was adopted as the best fit model for PPR. The power regression model takes the form:

(3) $$\text{PPR}=a\;{\text{FR}}^{b}$$

where PPR = predicted permeation rate (ng·min^−1^) at 25 °C, FR = flow rate (mL·min^−1^), and *a* and *b* are curve fitting parameters. The best fit power regression equation models for each compound were thus derived as follows:

(4) $$\text{PPR}(\text{Benzene})=32.631{\text{FR}}^{0.5986}\ldots ({\text{R}}^{2}=0.09665)$$

(5) $$\text{PPR}(\text{Toluene})=11.048{\text{FR}}^{0.8166}\ldots ({\text{R}}^{2}=0.9569)$$

(6) $$\text{PPR}(\text{Ethylbenzene})=0.8392{\text{FR}}^{0.9561}\ldots ({\text{R}}^{2}=0.9444)$$

(7) $$\text{PPR}(m-\text{Xylene})=1.1694{\text{FR}}^{1.0745}\ldots ({\text{R}}^{2}=0.9819)$$

**Figure 4.**
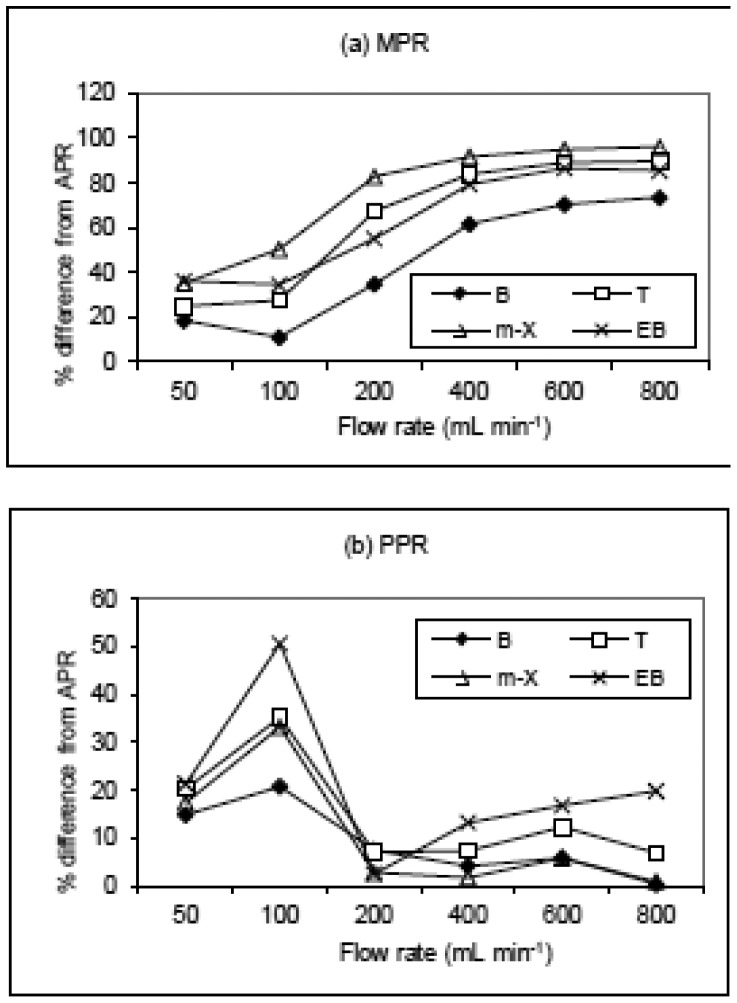
A plot of the percent difference (%) between (**a**) manufacturer's given PR (MPR); (**b**) predicted permeation rates (PPR) relative to the actual measured permeation rate APR as a function of flow rate (mL·min^−1^). Formulas: PD(MPR *vs.* APR) = [(MPR-APR)/APR] × 100 and PD(PPR *vs.* APR) = [(PPR-APR)/APR] × 100.

The plot of PPR points followed a curvilinear pattern proximate to that of APR (Figure 3). As shown in Figure 4, the enormity of bias (in terms of % difference values) between APR and PPR increased systematically with increasing FR. If the percent difference (PD) between APR and PPR is derived at 800 mL·min^−1^, the values tend to vary from 73.5 (benzene) to 96% (*m*-xylene). On the other hand, if this comparison is made at the lowest FR of 50 mL·min^−1^, the least PD values of 18.5 (benzene) to 36.2% (ethylbenzene) were computed.

### 3.4. Comparison between an Impinger and a Dynacalibrator System

To incubate PT devices at a very stable and accurate temperature, calibration gas standard generators are now commercially available but at a relatively high cost (e.g., Valco Instruments Co. Inc. (VICI), Schenkon, Switzerland). In our recent study, BTX standard gases were generated from PT devices at varying flow rates using a standard gas generator (dynacalibrator) equipped with more accurate temperature control system [7,8].

In order to assess the relative performance of the impinger system used in this study, the results of our analysis were compared with those derived using the dynacalibrator system in our recent study [7]. It confirms that the dynacalibrator with accurate temperature control generally maintains the low RSE values relative to impinger system. This implies the fact that the dynacalibrator can produce highly reproducible data due to reliability in its temperature control with high accuracy. In contrast, the impinger system used in this study is less accurate and stable in maintaining the constant temperature. This is because one should heat the water surrounding the impinger containing the PT device to control the permeation rate of the target compound. Using the dynacalibrator, RSE values measured at FR of 50 to 800 mL·min^−1^ were: 0.84 to 6.00% (B), 1.1 × 10^−14^ to 7.13% (T), and 1.75 to 8.75% (X) [7]. In contrast, the comparable data from the impinger system examined at FR of 800 and 50 mL·min^−1^ showed values in range of 2.36 to 7.69% (B), 3.59 to 9.28% (T), and 3.60 to 14.8% (X), respectively (Table 4).

As another means to assess the reliability of our impinger system, the compatibility between the two systems were also compared in terms of percent difference between the APR and PPR. The impinger data exhibited considerably low PD values (e.g., 14.8 and 20.8% for B, 20.3 and 35.2% for T, 21.4 and 50.6% for E, and 17.4 and 33.3% for X) at the two lower FR points (e.g., 50 and 100 mL·min^−1^), respectively. As such, the best compatibility was attained at moderately low FR (e.g., FR 50∼100 mL·min^−1^) (Table 4). These observed patterns are thus quite comparable with those of the dynacalibrator system in which the least PD values are attained at the lowest FR with generally smaller magnitude of PD across all FR ranges. For instance, the PD value of benzene varied from 1.2% at 20 mL·min^−1^ and 8.5% at 800 mL·min^−1^. As such, the use of such elaborate system as the dynacalibrator should be more reliable than the impinger system employed in this study. However, the results of this study confirm that the simplified gas standard generators such as our impinger system could also be used to yield fairly reliable results with the control of such major variables as temperature and flow rate.

### 3.5. Comparison between Different Systems for PT Applications

As mentioned in preceding sections, the PT-impinger system can be used to generate gas standard fairly constantly as the reproducibility of the system was maintained at acceptable range at most FR tested in terms of RSE values. Hence, FR can also play a key role in determining permeation rates (and consequently the generated gas standard concentration) of PT devices. The apparent role of air velocity (in addition to temperature) as a key parameter (as observed in this study) was also reported in some previous studies. An inverse relationship between PT-generated concentrations and FR was also observed in the study on the influence of carrier gas pressure on the permeation rates of NO_2_. Here, PT devices were selected as a ‘Hartmann & Braun’ CGP-NO_2_-test gas generator [15]. Our observation of a reduced bias at lower FR is however contrasting with the results of a VOC flux study by using wind tunnels and flux chambers, where a post-sampling correction factor was required [16].

According to Mitchell [17], practical departures from the theoretical performance of PT devices are the result of restrictions caused by various factors including operating temperature. McKinley [9] also reported that the emission rate of VOCs from a liquid-fed PT device typically varies about 10% per 1 °C. Hence, temperature control for stable operating conditions is crucial to generate calibration gas from a PT device at the desired concentration [18,19]. Based on this study, it is possible to infer that the PT-impinger system can be used to generate standard gases in a fairly consistent manner.

Moreover, our comparison of various approaches for gas standard generation suggests the need for the development of balancing equations for the proper use of PT devices in standard gas generation. Otherwise, if one relies on the manufacturer's guide, one may experience significantly large bias in the estimation of the produced gas standard concentration. A post-experiment correction for release rate in permeation tubes has also proven to reduce bias and improve the accuracy of the sulphur hexafluoride (SF_6_) tracer technique in deer [20]. These authors likewise recommended re-establishment of PT release rates to enable more accurate estimation of released compound concentration, when PT devices are used over an extended period.

## 4. Conclusions

This study tested the performance of an impinger system for the generation of calibration gas standards using PT devices for four aromatic VOCs (BTEX) across six different flow rates (50 to 800 mL·min^−1^). Results show that there are large discrepancies in concentration values between the manufacturer's formula and the actual concentrations. According to our experimental study, it was confirmed that the direct use of such manufacturer's formula for PT devices can actually lead to large biases in the generated concentration. It was found that PR values of BTEX generally vary dynamically as a function of FR (at a given temperature). As a clear curvilinear pattern with FR-APR plot is established, estimation of PR requires proper assessment of its relationship with FR. As such, derivation of equations for PPR at specific temperature should be considered a prerequisite for bias reduction between estimated and actually generated analyte concentrations.

Results of our study suggest that the impinger-PT system for gas standard generation should have yielded the least biased data below certain range of FR (e.g., <100 mL·min^−1^). Although the results of impinger derived data sets confirm the reliability of its application, the pattern of its bias (between APR and PPR) across varying flow rate tends to be distinguished greatly from those of a more deliberate system like dynacalibrator. In conclusion, the impinger system could be used as a low-cost and simple standard gas generation system provided that the relationship between PR and two experimental parameters (temperature and flow rate) are established for the adjustment of their concentration values.

## Figures and Tables

**Table 1.** Basic information of permeation tube used for the generation of target compounds (BTEX) used in this study.

**Compound full Name**	**Benzene**	**Toluene**	**Ethylbenzene**	**m-Xylene**
Abbreviation	B	T	E	X
Molecular Formula	C_6_H_6_	C_7_H_8_	C_8_H_10_	C_8_H_10_
Density (g·cm^−3^)	0.88	0.8669	0.8665	0.86
MW (g·mole^−1^)	78.11	92.14	106	106.2
CAS No.	71-43-2	108-88-3	100-41-4	108-38-3
Sales order No.	102597	102597	102597	102597
Part No.	100-160-1400-U70	100-183-1401-U80	100-141-1405-U100	100-114-1403-U100
Type	HE ^a^	HE ^a^	HE ^a^	HE ^a^
Total Length (cm)	19.5	21.8	14.1	14.9
Diameter (cm)	0.98	0.98	0.98	0.98
Permeation tube rate at T_o_ ^b^				
P_o_ (ng·min^−1^)	16,000 ± 15%	18,849 ± 15%	21,714 ± 15%	21,774 ± 15%
T_o_ (°C)	70	80	100	100
Permeation tube rate at 25 °C ^b^				
P_25_ (ng·min^−1^)	472	254	61.2	61.4
Molar constant at 25 °C (K) ^c^	0.313	0.27	0.26	0.23

a HE implies that the device is built for high emission rate;

b If a permeation rate (P_o_) is known at some reference temperature (T_o_), a new permeation rate (P_1_) at another temperature (T_1_) can be estimated as follows: log P_1_ = log P_o_ + 0.034 (T_1_–T_o_);

c Molar constant (K) = R·T/MW; where R = gas constant 0.082057 L·atm/mol·K; T = absolute temperature (°K); and MW = molecular weight (g·mole^−1^). K is included in the manufacturer's equation to calculate concentration (refer to Equation (2)).

**Table 2.** TD and GC-FID settings for the analysis of PT-generated standards of BTEX by sorbent tube method.

**GC-FID System (Varian 450- GC, USA)**			

Column: CP-WAX 52CB (Length: 60 m, ID: 0.25 mm, Film thickness: 0.25 μm, Chrompack)			
	Oven setting		Detector setting
Oven Temp:	50 °C (5 min)	Detector Temp:	240 °C
Oven rate:	6 °C·min^−1^	H_2_ flow:	30 mL·min^−1^
Max Oven Temp:	230 °C (5 min)	N_2_ flow:	29 mL·min^−1^
Total Time:	40 min	Air flow:	30 mL·min^−1^
Thermal desorber (Unity, Markes Ltd., UK)			

Sorbent tube/sample desorption temp.	300 °C	Valve temp	120 °C
Sorbent tube/sample desorption time	10 min	Transfer line temp	120 °C
Cold trap temp low	5 °C	Minimum pressure	10 psi
Cold trap temp high	300 °C	Split ratio	0
Cold trap hold time	5 min		

**Table 3.** Gas-based BTX calibration on the TD GC-FID system.

**Order**	**B**	**T**	**X**
(a) Information on analyte mass injected to the sorbent tubes (ng)			
1	9.58	11.3	9.58
2	38.3	45.2	38.3
3	95.8	113	95.8
4	192	226	192
5	479	565	479
6	958	1130	958
7	1437	1696	1437
(b) Detection limit (DL)			
ng	1.16	2.7	0.82
ppb	0.36	0.72	0.22
(c) Gas-based calibration results			
Slope	19,543	18,572	16,281
R^2^	1	0.999	0.9998
RSE (%)	0.68	0.16	0.67

**Table 4.** Experimental bias in estimating permeation rates of BTEX based on three different definitions for permeation rates ^a^.

		**Diluent (N_2_)**	**Concentration (ng·mL^−1^)**								**Permeation Rate (PR)**			**% Difference (PD)**	
					
Order	Compound	flow rate (mL·min^−1^)	MC ^b^	PC ^c^	AC						(ng·min^−1^)			from APR ^g^	
					R1	R2	R3	average	SD	RSE (%) ^d^	MPR	PPR ^e^	APR ^f^	MPR	PPR
1	Benzene	800	0.59	2.24	2.19	2.15	2.33	2.22	0.09	2.36	472	1,784	1,779	73.5	0.27
2		600	0.79	2.51	3.28	2.36	2.33	2.66	0.54	11.8	472	1,502	1,595	70.4	5.84
3		400	1.18	2.95	3.20	3.02	3.01	3.07	0.11	1.98	472	1,178	1,230	61.6	4.17
4		200	2.36	3.90	3.81	3.58	3.47	3.62	0.17	2.76	472	778	724	34.8	7.48
5		100	4.72	5.15	4.14	3.93	4.70	4.25	0.40	5.45	472	514	425	11.0	20.8
6		50	9.44	6.80	7.05	9.13	7.72	7.97	1.06	7.69	472	339	398	18.5	14.8

1	Toluene	800	0.32	3.25	2.93	2.92	3.25	3.04	0.19	3.59	254	2,594	2,429	89.5	6.79
2		600	0.42	3.43	5.26	3.24	3.19	3.90	1.18	17.5	254	2,051	2,337	89.1	12.3
3		400	0.64	3.69	4.16	3.94	3.82	3.97	0.17	2.52	254	1,473	1,589	84.0	7.33
4		200	1.27	4.19	4.22	3.83	3.67	3.91	0.28	4.19	254	836	781	67.5	7.04
5		100	2.54	4.76	3.42	3.28	3.83	3.51	0.29	4.69	254	475	351	27.6	35.2
6		50	5.09	5.40	5.71	7.88	6.71	6.77	1.09	9.28	254	270	338	24.8	20.3

1	Ethylbenzene	800	0.08	0.63	0.50	0.49	0.58	0.52	0.05	5.22	61.2	501	418	85.4	19.8
2		600	0.10	0.63	1.06	0.62	0.60	0.76	0.26	19.73	61.2	380	457	86.6	16.8
3		400	0.15	0.64	0.79	0.73	0.71	0.74	0.04	3.23	61.2	258	297	79.4	13.2
4		200	0.31	0.66	0.74	0.67	0.64	0.68	0.05	4.37	61.2	133	136	55.1	2.50
5		100	0.61	0.69	0.45	0.42	0.49	0.46	0.04	4.80	61.2	68.6	45.5	34.5	50.6
6		50	1.22	0.71	0.73	1.04	0.92	0.90	0.15	9.92	61.2	35.3	44.9	36.2	21.4

1	m-Xylene	800	0.08	1.92	1.84	1.83	2.05	1.91	0.12	3.60	61.4	1,539	1,526	96.0	0.85
2		600	0.10	1.88	2.86	1.60	1.53	2.00	0.75	21.6	61.4	1,130	1,200	94.9	5.79
3		400	0.15	1.83	1.98	1.82	1.78	1.86	0.10	3.22	61.4	731	744	91.7	1.72
4		200	0.31	1.74	2.12	1.68	1.56	1.78	0.29	9.52	61.4	347	357	82.8	2.72
5		100	0.61	1.65	1.12	1.00	1.59	1.24	0.31	14.41	61.4	165	124	50.4	33.3
6		50	1.23	1.57	1.45	2.39	1.85	1.89	0.47	14.47	61.4	78.3	94.7	35.2	17.4

a Acronyms: MC = concentration simply derived using manufacturer's equation; PC = concentration predicted by the experimentally derived equation from this study; AC = actually measured concentration from our experiment; MPR = given permeation rate calculated based on the manufacturer's equation; PPR = predicted permeation rate derived from our experiment; and APR = actual measured permeation rate;

b Given concentration of each compound is calculated based on the manufacturer's permeation tube rate (ppm) = KxP/F; where: P = permeation rate (ng·min^−1^); F = dilution flow (mL·min^−1^); K (compound molar constant in g·L^−1^) = R·T/MW (R = gas constant 0.082057 L·atm/mol·K; T = 273 + actual temperature; and MW = molecular weight of gas in g·mole^−1^);

c Equation for predicted concentration in ng·mL^−1^ (Y) for each compound was derived using power regression with flow rate (mL·min^−1^) as independent variable X: Y_benzene_ = 32.631 × ^−0.401^; Y_toluene_ = 11.048 × ^−0.183^; Y_m-xylene_ = 1.1694 × ^0.0745^; Y_ethylbenzene_ = 0.8392 × ^−0.044^;

d Percent relative standard error (RSE: %) = SE/average × 100, where standard error (SE) = standard deviation / $\sqrt{n}$;

e Equation for predicted permeation rate in ng·min^−1^ (Y) for each compound was derived using power regression with flow rate (mL·min^−1^) as independent variable X: Y_benzene_ = 32.631^0.5986^; Y_toluene_ = 11.048 × ^0.8166^; Y_m-xylene_ = 1.1694 × ^1.0745^; Y_ethylbenzene_ = 0.8392 × ^0.9561^;

f Equation for measured permeation rate (PR in ng·min^−1^) = CxF/K where C = measured concentration in ng·mL^−1^; F = flow rate in mL·min^−1^; and K compound molar constant in g·L^−1^;

g Percent difference (PD: %) = (predicted-measured)/measured × 100.

## Abbreviations

AC: Measured concentrations of BTEX from PT-generated samples
APR: Actual PR based on the GC-based quantification of target compounds
B: Benzene
E: Ethylbenzene
FR: Flow rate
MC: Expected concentrations of PT-generated gases computed using the manufacturer-published equation
MPR: Manufacturer's PR
PC: predicted concentrations
PPR: Predicted PR
PT: Permeation tube
T: Toluene
X: *m*-Xylene
